# No Difference in Overall Survival and Non-Breast Cancer Deaths after Partial Breast Radiotherapy Compared to Whole Breast Radiotherapy—A Meta-Analysis of Randomized Trials

**DOI:** 10.3390/cancers12082309

**Published:** 2020-08-17

**Authors:** Jan Haussmann, Wilfried Budach, Stefanie Corradini, David Krug, Balint Tamaskovics, Edwin Bölke, Freddy-Joel Djiepmo-Njanang, Ioannis Simiantonakis, Kai Kammers, Christiane Matuschek

**Affiliations:** 1 Department of Radiation Oncology, Heinrich Heine University, 40225 Duesseldorf, Germany; Jan.Haussmann@med.uni-duesseldorf.de (J.H.); Wilfried.Budach@med.uni-duesseldorf.de (W.B.); Balint.Tamaskovics@med.uni-duesseldorf.de (B.T.); FreddyJoel.DjiepmoNjanang@med.uni-duesseldorf.de (F.-J.D.-N.); Ioannis.Simiantonakis@med.uni-duesseldorf.de (I.S.); matuschek@med.uni-duesseldorf.de (C.M.); 2 Department of Radiation Oncology, LMU University, 81377 Munich, Germany; Stefanie.corradini@med.uni-muenchen.de; 3 Department of Radiation Oncology, University Hospital Schleswig-Holstein, 24105 Kiel, Germany; David.Krug@uksh.de; 4 Division of Biostatistics and Bioinformatics, Department of Oncology, The Sidney Kimmel Comprehensive Cancer Center at Johns Hopkins, The Johns Hopkins University School of Medicine, Baltimore, MD 21205, USA; kai.kammers@jhu.edu

**Keywords:** cancer, investigation, study, radiation therapy, randomized

## Abstract

Purpose/objective: Adjuvant radiotherapy after breast conserving surgery is the standard approach in early stage breast cancer. However, the extent of breast tissue that has to be targeted with radiation has not been determined yet. Traditionally, the whole breast was covered by two opposing tangential beams. Several randomized trials have tested partial breast irradiation (PBI) compared to whole breast irradiation (WBI) using different radiation techniques. There is evidence from randomized trials that PBI might result in lower mortality rates compared to WBI. We aimed to reassess this question using current data from randomized trials. Material/methods: We performed a systematic literature review searching for randomized trials comparing WBI and PBI in early stage breast cancer with publication dates after 2009. The meta-analysis was performed using the published event rates and the effect sizes for overall survival (OS), breast cancer-specific survival (BCSS), and non-breast cancer death (NBCD) as investigated endpoints. Analysis of subgroups using different radiation techniques was intended. We used hazard ratios (HR) and risk differences (RD) to estimate pooled effect sizes. Statistical analysis was performed using the inverse variance heterogeneity model. Results: We identified eleven studies randomizing between PBI and WBI. We did not find significant differences in OS (*n* = 14,070; HR = 1.02; CI-95%: 0.89–1.16; *p* = 0.810, and *n* = 15,203; RD = −0.001; CI-95%: −0.008–0.006; *p* = 0.785) and BCSS (*n* = 15,203; RD = 0.001; CI-95%: −0.002–0.005; *p* = 0.463). PBI also did not result in a significant decrease of NBCD (*n* = 15,203; RD = −0.003; CI-95%: −0.010–0.003; *p* = 0.349). A subgroup analysis by radiation technique also did not point to any detectable differences. Conclusion: In contrast to a previous assessment of mortality, we could not find a detrimental effect of WBI on OS or NBCD. A longer follow-up might be necessary to fully assess the long-term mortality effects of PBI compared to WBI.

## 1. Introduction

The standard treatment of early stage breast cancer is breast conservation surgery followed by whole breast irradiation (WBI) and appropriate systemic therapy. This regime has been shown to be equivalent to mastectomy in terms of overall survival in numerous randomized trials [1,2,3,4,5,6,7]. The analysis of local recurrences in the treated breast suggested that the majority occur at the original tumor location [8,9,10,11]. This led to the hypothesis that adjuvant treatment of the tumor bed might be equally effective to whole breast radiotherapy and potentially associated with less side effects.

Multiple randomized trials addressing this question, using a multitude of techniques, have been conducted [12,13,14,15,16,17]. A pooled analysis of randomized trials reported a reduced non-breast cancer survival (NBCD) and overall survival (OS) rate in patients undergoing WBI as compared to patients treated with partial breast irradiation (PBI) [18]. This result was surprising, given the relatively short follow-up of the majority of the included trials. Over the last decade, it has been increasingly accepted that WBI contributes in a dose dependent way to major coronary events [19,20] and other secondary malignancies, including lung cancer [20]. It is hypothesized that PBI might lower the occurrence of these late adverse events by reducing the dose to the corresponding organs at risk.

We aimed to reassess this question and include recently published trials and trials with longer follow-up.

## 2. Material and Methods

On 10 April 2020, we performed a literature review according to the published PRISMA guideline [21]. We searched the MEDLINE as well as the EMBASE and EBM review platforms. Further, we screened the major meetings for published abstracts. The chosen keywords were (“radiation therapy” or “radiotherapy” or “irradiation”) AND (“breast cancer” or “carcinoma of the breast”) AND (“partial” or “targeted”) AND (“randomized” OR “randomised” OR “randomly”).

We included randomized controlled trials that investigated patients suffering from invasive breast cancer or carcinoma in situ comparing PBI to WBI. Trials had to be published after 31 December 2009, in order to include comparable modern techniques. We excluded trials that solely included carcinoma in situ patients.

We extracted the provided hazard ratios and event numbers from the identified trials to estimate the effect sizes, comparing WBI to PBI in the endpoints of overall survival (OS) as well as breast cancer-specific survival (BCSS), non-breast cancer death (NBCD), and cardiac deaths (CD). The definition of the analyzed endpoints was adopted from the published trials. When no specific event numbers were given in the publications, we calculated the events with the assumption that total mortality events equal the sum of deaths from breast cancers plus the non-breast cancer deaths. When no hazard ratios were reported, we estimated the hazard ratio and their corresponding 95% confidence interval by reconstructing all events from the published survival curves or using the method published by Parmar and Tierney [22,23]. When hazard ratios were neither reported nor estimable, we used the absolute number of events and calculated the risk differences and the corresponding confidence interval.

We used the inverse variance of heterogeneity model (ivhet) to estimate the pooled effect sizes. This method favors larger trials, uses a more conservative estimation of the confidence limits, and produces lesser observed variances compared to the random effects model [24]. Zero event correction was applied, where appropriated [25]. *p*-values below the threshold of 0.05 were considered statistically significant.

The measurement of heterogeneity within the meta-analysis was obtained with Cochran’s Q-test with the corresponding *p*-values [26,27]. Further, we also described the I^2^ statistics where we defined values above 25% as considerable heterogeneity [28]. Funnel plots were created for a visual analysis of publication bias. Statistical analysis was performed using the Microsoft Excel add-in MetaXL 5.3 (EpiGear International, Sunrise Beach, Australia). Plots were created using Microsoft Excel for Microsoft Office 365 Pro Plus (Redmond, Washington, WA, USA).

In order to compare different techniques, we pooled the results of each one of external beam radiation, intraoperative radiotherapy using electrons or photons as well any brachytherapy, including single- or multicatheter based approaches. We recognize that this approach ignores the detailed differences between the individual techniques, which each have their own advantages. However, creating a subgroup for any techniques makes a general comparison impossible and ignores the basic approaches to each treatment.

As the TARGIT-group has recently updated the long-term results of the postpathology or delayed treatment subgroup, we decided to split the trial into the original prepathology group with shorter follow-up and postpathology group with longer follow-up, as we felt that this approach allowed the most appropriate estimation of the desired comparison.

The assessments of other oncological endpoints as well as adverse events were not the aim of this investigation and will be reported separately.

## 3. Results

**Figure 1:**
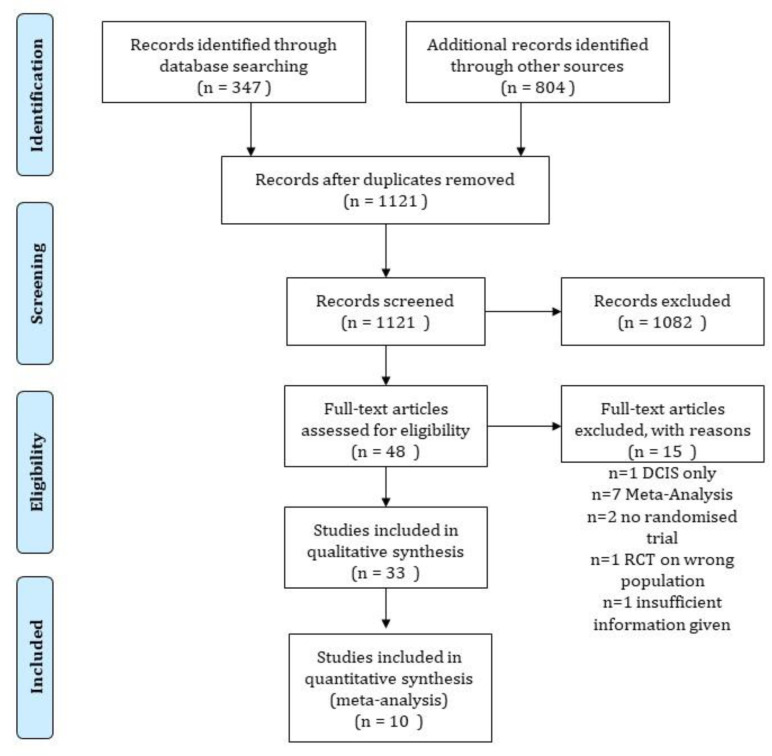
Consort diagram showing the results of the literature review according to the PRISMA guidelines.

The literature search as shown in Figure 1 identified eleven studies randomizing an overall number of 15,438 patients. A total of ten trials reported event numbers for the endpoints of OS, BCSS, and NBCD. Eight trials allowed an estimation of the hazard ratios for OS.

An overview of the included studies is given in Table 1. 

The included trial populations consisted mainly of node negative, hormone receptor positive, low-risk breast cancer patients. There were 1527 (10.0%; range: 0–24%; median: 0%) patients with ductal carcinoma in situ (DCIS) and 12640 participants with hormone receptor positive disease included (82.7%; range: 56–96%; median: 88.8%). Concerning high risk factors, undifferentiated grading (grade 3) was present in 2422 patients (15.8%; range: 0–27%; median: 11.3%) and positive lymph nodes in 1377 participants (9.0%; range: 0–26%; median: 3.0%). There were 2566 (16.8%) women below 50 years included (range: 0–38%; median: 12.0%). Between 3% and 29% received chemotherapy as part of their treatment (n = 2213; 15.4%; median: 10.7%) and 6941 took endocrine therapy (62.7%; range 49–90%; median: 67.3%).

In terms of radiation technique, six studies used photon or electron based PBI, three studies utilized intraoperative radiotherapy (IORT), whereas three trials investigated interstitial brachytherapy using single- and multicatheter based methods. The PBI schedules consisted of conventionally fractionated RT, once daily hypofractionated RT (QD RT) as well as accelerated hypofractionated RT schedules (twice daily/BID RT). There was no evidence of publication bias according to the funnel plots (not shown). Median follow-up of the included trials ranged between 2.4 and 10.5 years.

**Figure 2:**
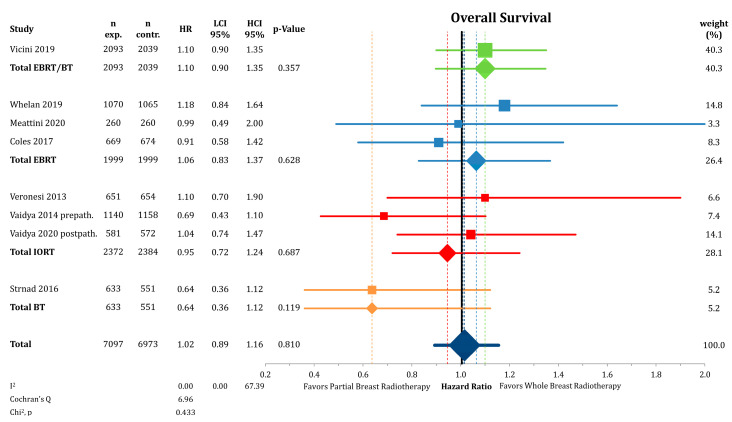
Comparison of overall survival by hazard ratios between partial- and whole breast radiation using a forest plot and the inverse variance heterogeneity model. Trials are grouped by radiation technique. Quadrats and diamonds represent individual trials and pooled effect sizes with corresponding 95% confidence intervals.

Figure 2 shows the analysis of OS between PBI and WBI, which was not statistically different between the groups (*n* = 14,070; HR = 1.02; CI-95%: 0.89–1.16; *p* = 0.810). There was no detectable heterogeneity (I^2^ = 0.00). We found no significant differences in the subgroup analysis by radiation technique.

**Figure 3:**
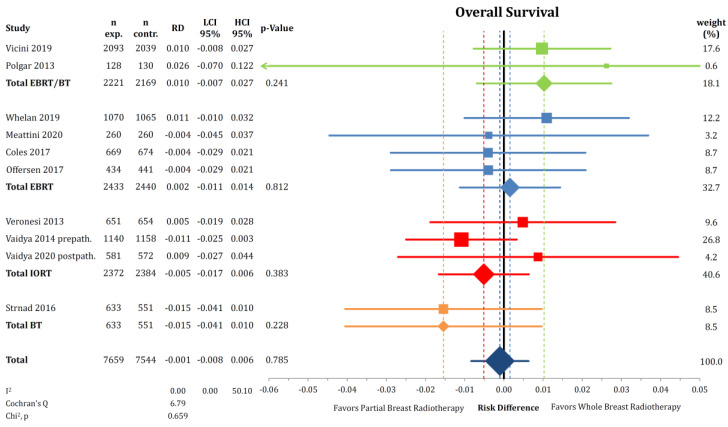
Comparison of overall survival by risk difference between partial- and whole breast radiation using a forest plot and the inverse variance heterogeneity model. Trials are grouped by radiation technique. Quadrats and diamonds represent individual trials and pooled effect sizes with corresponding 95% confidence intervals.

We obtained similar results regarding OS when analyzing risk differences, as depicted in Figure 3. The absolute difference in the proportion of patients dying between the groups was 0.1% (*n* = 15,203; RD = −0.001; CI-95%: −0.008–0.006; *p* = 0.785) without any noticeable heterogeneity (I^2^ = 0.00). In absolute terms, the percentages of patients alive in the trials were 93.17% with PBI and 93.40% with WBI in cumulative numbers at the last reported follow-up point using the raw data.

**Figure 4:**
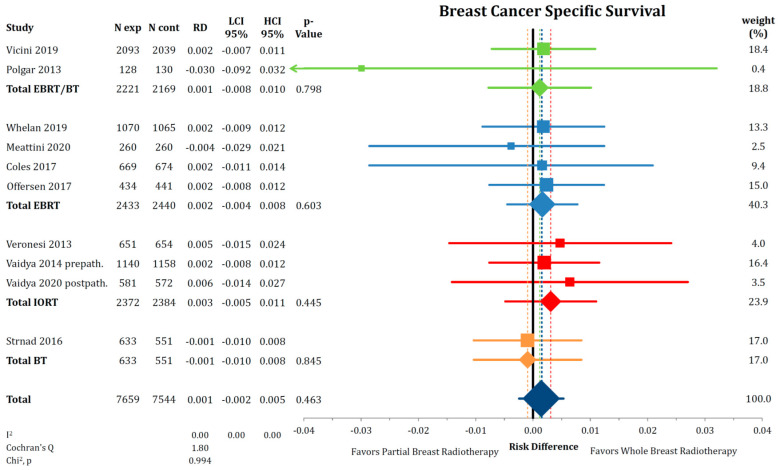
Comparison of breast cancer-specific survival by risk difference between partial- and whole breast radiation using a forest plot and the inverse variance heterogeneity model. Trials are grouped by radiation technique. Quadrats and diamonds represent individual trials and pooled effect sizes with corresponding 95% confidence intervals.

**Figure 5:**
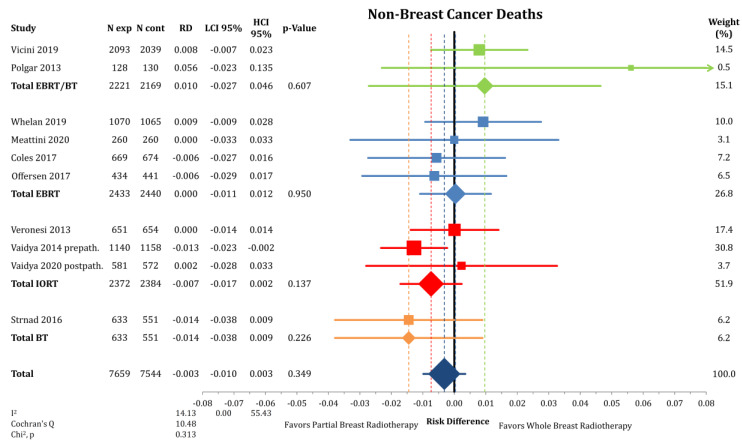
Comparison of non-breast cancer death by risk difference between partial- and whole breast radiation by a forest plot. Trials are grouped by radiation technique. Quadrats and diamonds represent individual trials and pooled effect sizes with corresponding 95% confidence intervals.

Breast cancer-specific survival was also not different between PBI and WBI with a risk difference of 0.1% (*n* = 15,203; RD = 0.001; CI-95%: −0.002–0.005; *p* = 0.463) without any detectable heterogeneity (I^2^ = 0.00) (Figure 4). The cumulative incidence of death from breast cancer was 2.0% after PBI and 1.9% after WBI. Additionally, mortality due to other causes than breast cancer (NBCD) was not different in the PBI group compared to WBI with a risk difference of 0.3% (*n* = 15,203; RD = −0.003; CI-95%: −0.010–0.003; *p* = 0.349) (Figure 5). The analysis revealed no heterogeneity between the trials (I^2^ = 14.13). Cumulatively, 4.8% in the PBI and 4.7% in the WBI arms of the included patients died from other causes than breast cancer.

**Figure 6:**
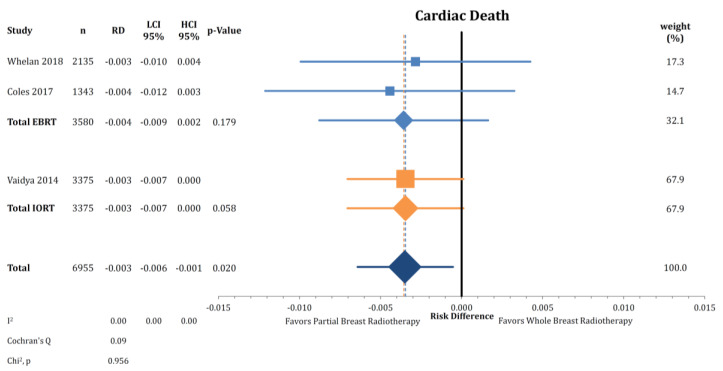
Comparison of cardiac death by risk difference between partial- and whole breast radiation using a forest plot. Trials are grouped by radiation technique. Quadrats and diamonds represent individual trials and pooled effect sizes with corresponding 95% confidence intervals.

Three trials further reported on cardiac death as part of NBCD (Figure 6). Pooling the risk differences, we obtained a significant reduction in cardiac deaths of 0.3% in patients treated with PBI (*n* = 6955; RD = −0.003; CI-95%: −0.006–0.001; *p* = 0.020). The test for heterogeneity showed no significant effect (I^2^ = 0.00).

## 4. Discussion

In women with low-risk breast cancer, partial breast irradiation, in comparison to whole breast irradiation, does not result in a reduction in overall or non-breast cancer mortality. This result stands in contrast to the previously published analysis by Vaidya and colleagues, which reported a “small yet statistically and clinically significant difference between PBI and WBI favoring PBI” in OS and NBCD [18]. Is this paper to be regarded as an updated version of the previous meta-analysis or are there substantial differences explaining the different conclusions? The most notable difference between the meta-analyses is the enlarged patient numbers included as well as the lengthened follow-up time in our investigation. The previous analysis described the outcome data of 4231 to 4489 patients in the investigated endpoints in four to five trials, with only one trial following their patients longer than a median of more than 5–6 years. In contrast, this analysis included 8 to 10 trials with more than three times the size, with up to 15,212 patients treated within the studies reporting on a median follow-up longer than eight years in five trials.

Both analyses are very similar regarding the statistical outcome measure (risk differences) and the trial inclusion criteria. However, the model used to pool and compare the effect sizes was different, as we utilized the inverse variance heterogeneity model instead of the random and fixed effect models. The reasons for this are described above. Nonetheless, replacing the ivhet with the random effects model in our analysis did not change the forthcoming effect sizes (results not shown) and thus maintained the interpretation of the results.

The analysis by Vaidya et al. included more patients treated with brachytherapy or intraoperative radiotherapy (~87%) [18]. Does this varying distribution of PBI techniques explain the different outcome? Our results do not support this interpretation, as the estimation of heterogeneity did not suggest an effect by different trials. Three of the included trials (about 24% of trial population) predominantly applied WBI in a hypofractionated, accelerated schedule [16,32,55]. This treatment schedule has been shown to cause lower rates of acute as well as late adverse events, which could have introduced some heterogeneity in the control arms. The pooled analysis of the START trials described numerically lower event numbers in the trials after 10 years of follow-up [56]. If the estimated alpha/beta ratios for late cardiac events are estimated correctly, one could expect lower long-term cardiac morbidity after shorter course radiation treatment.

A given explanation for a hypothesized reduced mortality is that with PBI, especially with focal therapy (BT, IORT), lower doses are applied to the heart. This might translate to fewer late cardiac adverse events like ischemic heart disease, chronic heart failure, or cardiac arrhythmia. This is supported by the hypothesis-generating analysis of cardiac death, which demonstrated that PBI resulted in fewer cardiac deaths. However, we detected no variation between EBRT and IORT with numerically similar risk differences (Figure 6), which leads to the conclusion that the effect may be independent of the applied technique. Given the extensive efforts in modern radiation oncology of keeping the heart doses as low as possible using different techniques, like cardiac shielding and deep-inspiration breath hold (DIBH), we anticipate the difference to be lower than estimated here [57]. Unfortunately, an analysis of NBCD or cardiac death divided by laterality of breast cancer was not possible. Early results of a randomized trial of radiotherapy, including the internal mammary nodes, using DIBH compared to free-breathing in patients with left-sided breast cancer found reduced cardiac doses and improved left ventricular ejection fraction in the DIBH group [58,59]. Classically, it has always been assumed that the cardiovascular harm from tangential radiation therapy manifests at 10+ years of follow-up [60]. We would, however, caution the interpretation that PBI reduces the cardiac death rate compared to WBI. Firstly, the trials that actually reported on cardiac deaths are very limited (*n* = 3). Secondly, the attribution of a cause of death is often difficult and subject to possible inconsistency. Lastly, cardiac deaths are a substantial component of the non-breast cancer mortality events, which demonstrated no difference between PBI and WBI.

Possible shortcomings of this analysis include the still relatively limited median follow-up between 5 and 10 years. The included data are publication-based data, rather than individual patient-based data (IPD), which would be generally desirable. Further, a comprehensive assessment of the different PBI techniques was not possible, as the NSABP B-39 and Budapest trials did not report separately on the effects of EBRT and BT in the investigated endpoints.

How does this analysis compare to other publications? The meta-analysis by Hickey et al. also reports no effect of PBI on OS and BCSS, similar to our analysis [61]. In contrast, in the paper published by Korzets and colleagues in 2019, PBI led to reduced non-breast cancer death (OR = 0.55; CI-95%: 0.41–0.73) with a trend for improved overall survival (OR = 0.84; CI-95%: 0.71–1.01) [23]. However, the investigation is limited by the lack of the inclusion of the RAPID and NSABP B-39 trials in the evaluation of NBCD as well as missing current data from RAPID, NSABP B-39, and Florence trials in the OS assessment. It is very possible that the long-term outcome data comparing PBI and WBI will show a clearer picture of what might be starting to transpire in the presented data and also makes common sense: PBI might be superior in terms of toxic effects of radiation, but might be slightly inferior in cancer control compared to WBI.

The included trials differ substantially in terms of included subgroups, as the inclusion criteria range from DCIS up to patients with multiple positive lymph nodes and triple negative disease. Current guidelines encourage the usage of different RT techniques and advise the selection of low-risk patients when using partial breast treatments [62,63,64,65,66]. In this analysis, we detected no difference of the PBI techniques, leading to the conclusion that all used treatment strategies appear to provide similar survival results.

## 5. Conclusions

In summary, our meta-analysis demonstrated that patients treated in randomized trials comparing partial to whole breast irradiation did not show any differences in mortality.

## Figures and Tables

**Table 1:** Overview of the included trials.

Study	Synonym	Additional Publications	Y Trial	FU	N Total	Med. Age	Stat. Setting	Prim. EP	Pop	Strat.	PBI Technique	PBI Dose	WBI Dose	G3	DCIS	N+	HR+	Her2+	CTx	ET	Boost
Vicini 2019 [29]	NSABP B-39	Vicini et al. 2019 [30] White et al. 2019 [31]	2005–2013	10.2	4216	54	Equiv.	IBTR	IBC or DCIS; T < 3 cm, ≤N1; R0; >18y	Stage, Menopausal, ER, CTx	3DCRT, single- and multicath. BT	34/3.4; 38.5/3.85 10x in 5-8d	50/2; 50.4/1.8; opt. Boost	26%	24%	10%	81%	n.r.	29%	n.r.	80%
Whelan 2019 [32]	RAPID	Olivotto et al. 2013 [33] Peterson et al. 2015 [34] Whelan et al. 2019 [35]	02/2006–07/2011	8.6	2135	61	noninf	IBTR	IBC or DCIS; T < 3cm;R0; N0; >40 y; unifocal	Age > < 50; Histology, T > < 1.5 cm; ER, Center	3DCRTIMRT	38.5/3.85 BID in 5-8d	50/2; 42.5/2.66 + opt. Boost	16%	18%	0%	84%	6%	13%	55%	21%
Meattini 2020 [36]	Florence	Livi et al. 2010 [37] Livi et al. 2015 [38] Meattini et al. 2017 [39]	03/2005–06/2013	10	520	n.r.	noninf	IBTR	IBC or DCIS; T < 2.5 cm; >40 y; BCS +	None	IMRT	30/6	50/2 + opt. 10/2	11.4%	11%	10%	96%	4%	4%	61%	n.r.
Veronesi 2013 [13]	ELIOT		11/2000–12/2007	5.8	1305	nr	noninf	IBTR	IBC; T < 2.5 cm; R0; 48–75y; unifocal	T < 1–1.4 > cm	IORT e-	21/21	50/2 + opt. 10/2	20.9%	0%	27%	91%	3%	8%	88%	n.r.
Vaidya 2014 [12]	TARGIT-A	Vaidya et al. 2010 [40] Andersen et al. 2012 [41] Sperk et al. 2012 [42] Welzel et al. 2013 [43] Keshtgar et al. 2013 [44] Corica et al. 2016 [45] Corica et al. 2018 [46]	03/2000–06/2012	2.4	3375	Mean 63	noninf	IBTR	IDC; T < 2.5 cm; R0; >45 y; unifocal	Center, timing	IORT x	20/20	n.r.	14.2%	0%	16%	92%	12%	12%	66%	38%
Vaidya 2020 [47]	TARGIT-A postpathology		03/2000–06/2012	9	1153	Mean 63	noninf	IBTR	IDC; T < 2.5cm; R0; >45y; unifocal	Center, timing	IORT x	20/20	n.r.	6%	3%	5%	98%	6%	4%	87%	n.r.
Strnad 2016 [15]	GEC Estro	Polgar et al. 2017 [48] Schäfer et al. 2018 [49]	04/2004–07/2009	6.6	1328	62	noninf	IBTR	IBC or DCIS; T < 3 cm; R0; N0; >40 y; BCS +	Center, Menopausal, stage	Multicath. BT	32/4; 30.3/4.3 or PDR	50/2; 50.4/1.8; opt. Boost	8.3%	5%	6%	95%	n.r.	11%	90%	98%
Coles 2017 [16]	Import low	Bhattacharya et al. 2019 [50] Bhattacharya et al. 2019 [51] Bhattacharya et al. 2019 [52]	05/2007–10/2010	6	1343	62	noninf	IBTR	IDC; T < 3 cm; >50 y; pN0-1	Center	3DCRT	40/2.67 QD	40/2.67	9.7%	0%	3%	95%	4%	5%	80%	n.r.
Polgar 2013 [14]	Budapest	Polgar et al. 2004 [53] Polgar et al. 2007 [54] Polgar et al. 2017 [48]	1998–2004	10.5	258	Mean 59	noninf	LR	IBC; T < 2 cm; N0; R0; G1-2; unifocal	None	Multicath. BT 3DCRT	BT:36.4/5.2 BID; e-:50/2 QD	50/2 + opt. 16/2	0.0%	0%	5%	88%	n.r.	3%	99%	0.8%
Offersen 2017 [55]	DBCG PBI		2009–2016	3	882	66	noninf	Breast Induration 3y	IBC, T1, R0, >60 y, G1-2, HER2-, pN0	Center, ET	3DCRT	40/2.66 QD	40/2.66	<1.0%	0%	0%	100%	0%	n.r.	80%	n.r.

## Abbreviations

3DCRT	3D conventional radiation therapy
BCS	breast conserving surgery
BID	twice daily
BT	brachytherapy
CTx	chemotherapy
DCIS	ductal carcinoma in situ
e-	electrons
EBRT	external beam radiotherapy
EP	endpoint
ER	estrogen receptor
ET	endocrine therapy
FU	follow-up
IBC	invasive breast cancer
IDC	invasive ductal cancer
IBTR	in breast tumor recurrence
IMRT	intensity modulated radiation therapy
IORT	intraoperative radiotherapy
HR+	hormone receptor positive
LR	local recurrence
Med.	median
n	number
N+	nodal positive
Noninf	non-inferiority
n.r.	not reported
PBI	partial breast irradiation
Pop	Population
QD	once daily
RT	radiotherapy
q.o.d.	every other day
Stat.	statistical
Strat.	stratification
WBI	whole breast irradiation
x	photons
y	years

## References

[1] Fisher B., Anderson S., Bryant J., Margolese R.G., Deutsch M., Fisher E.R., Jeong J.H., Wolmark N. (2002). Twenty-year follow-up of a randomized trial comparing total mastectomy, lumpectomy, and lumpectomy plus irradiation for the treatment of invasive breast cancer. N. Engl. J. Med..

[2] Veronesi U., Cascinelli N., Mariani L., Greco M., Saccozzi R., Luini A., Aguilar M., Marubini E. (2002). Twenty-year follow-up of a randomized study comparing breast-conserving surgery with radical mastectomy for early breast cancer. N. Engl. J. Med..

[3] Poggi M.M., Danforth D.N., Sciuto L.C., Smith S.L., Steinberg S.M., Liewehr D.J., Menard C., Lippman M.E., Lichter A.S., Altemus R.M. (2003). Eighteen-year results in the treatment of early breast carcinoma with mastectomy versus breast conservation therapy: The National Cancer Institute Randomized Trial. Cancer.

[4] Arriagada R., Le M.G., Rochard F., Contesso G. (1996). Conservative treatment versus mastectomy in early breast cancer: Patterns of failure with 15 years of follow-up data. Institut Gustave-Roussy Breast Cancer Group. J. Clin. Oncol..

[5] Van Dongen J.A., Voogd A.C., Fentiman I.S., Legrand C., Sylvester R.J., Tong D., van der Schueren E., Helle P.A., van Zijl K., Bartelink H. (2000). Long-term results of a randomized trial comparing breast-conserving therapy with mastectomy: European Organization for Research and Treatment of Cancer 10801 trial. J. Natl. Cancer Inst..

[6] Skandarajah A.R., Mann G.B. (2010). Do All Patients Require Radiotherapy after Breast-Conserving Surgery?. Cancers.

[7] Corradini S., Reitz D., Pazos M., Schönecker S., Braun M., Harbeck N., Matuschek C., Bölke E., Ganswindt U., Alongi F. (2019). Mastectomy or Breast-Conserving Therapy for Early Breast Cancer in Real-Life Clinical Practice: Outcome Comparison of 7565 Cases. Cancers.

[8] Smith T.E., Lee D., Turner B.C., Carter D., Haffty B.G. (2000). True recurrence vs. new primary ipsilateral breast tumor relapse: An analysis of clinical and pathologic differences and their implications in natural history, prognoses, and therapeutic management. Int. J. Radiat. Oncol. Biol. Phys..

[9] Gujral D.M., Sumo G., Owen J.R., Ashton A., Bliss J.M., Haviland J., Yarnold J.R. (2011). Ipsilateral breast tumor relapse: Local recurrence versus new primary tumor and the effect of whole-breast radiotherapy on the rate of new primaries. Int. J. Radiat. Oncol. Biol. Phys..

[10] Vaidya J.S., Vyas J.J., Chinoy R.F., Merchant N., Sharma O.P., Mittra I. (1996). Multicentricity of breast cancer: Whole-organ analysis and clinical implications. Br. J. Cancer.

[11] Kaiser J., Reitsamer R., Kopp P., Gaisberger C., Kopp M., Fischer T., Zehentmayr F., Sedlmayer F., Fastner G. (2018). Intraoperative Electron Radiotherapy (IOERT) in the Treatment of Primary Breast Cancer. Breast Care.

[12] Vaidya J.S., Wenz F., Bulsara M., Tobias J.S., Joseph D.J., Keshtgar M., Flyger H.L., Massarut S., Alvarado M., Saunders C. (2014). Risk-adapted targeted intraoperative radiotherapy versus whole-breast radiotherapy for breast cancer: 5-year results for local control and overall survival from the TARGIT-A randomised trial. Lancet.

[13] Veronesi U., Orecchia R., Maisonneuve P., Viale G., Rotmensz N., Sangalli C., Luini A., Veronesi P., Galimberti V., Zurrida S. (2013). Intraoperative radiotherapy versus external radiotherapy for early breast cancer (ELIOT): A randomised controlled equivalence trial. Lancet Oncol..

[14] Polgar C., Fodor J., Major T., Sulyok Z., Kasler M. (2013). Breast-conserving therapy with partial or whole breast irradiation: Ten-year results of the Budapest randomized trial. Radiother. Oncol..

[15] Strnad V., Ott O.J., Hildebrandt G., Kauer-Dorner D., Knauerhase H., Major T., Lyczek J., Guinot J.L., Dunst J., Gutierrez Miguelez C. (2016). 5-year results of accelerated partial breast irradiation using sole interstitial multicatheter brachytherapy versus whole-breast irradiation with boost after breast-conserving surgery for low-risk invasive and in-situ carcinoma of the female breast: A randomised, phase 3, non-inferiority trial. Lancet.

[16] Coles C.E., Griffin C.L., Kirby A.M., Titley J., Agrawal R.K., Alhasso A., Bhattacharya I.S., Brunt A.M., Ciurlionis L., Chan C. (2017). Partial-breast radiotherapy after breast conservation surgery for patients with early breast cancer (UK IMPORT LOW trial): 5-year results from a multicentre, randomised, controlled, phase 3, non-inferiority trial. Lancet.

[17] Ovalle V., Strom E.A., Shaitelman S., Hoffman K., Amos R., Perkins G., Tereffe W., Smith B.D., Stauder M., Woodward W. (2018). Proton Partial Breast Irradiation: Detailed Description of Acute Clinico-Radiologic Effects. Cancers.

[18] Vaidya J.S., Bulsara M., Wenz F., Coombs N., Singer J., Ebbs S., Massarut S., Saunders C., Douek M., Williams N.R. (2016). Reduced Mortality With Partial-Breast Irradiation for Early Breast Cancer: A Meta-Analysis of Randomized Trials. Int. J. Radiat. Oncol. Biol. Phys..

[19] Darby S.C., Ewertz M., McGale P., Bennet A.M., Blom-Goldman U., Brønnum D., Correa C., Cutter D., Gagliardi G., Gigante B. (2013). Risk of Ischemic Heart Disease in Women after Radiotherapy for Breast Cancer. N. Engl. J. Med..

[20] Taylor C., Correa C., Duane F.K., Aznar M.C., Anderson S.J., Bergh J., Dodwell D., Ewertz M., Gray R., Jagsi R. (2017). Estimating the Risks of Breast Cancer Radiotherapy: Evidence From Modern Radiation Doses to the Lungs and Heart and From Previous Randomized Trials. J. Clin. Oncol..

[21] Moher D., Liberati A., Tetzlaff J., Altman D.G., Group P. (2009). Preferred reporting items for systematic reviews and meta-analyses: The PRISMA statement. BMJ.

[22] Parmar M.K., Torri V., Stewart L. (1998). Extracting summary statistics to perform meta-analyses of the published literature for survival endpoints. Stat. Med..

[23] Tierney J.F., Stewart L.A., Ghersi D., Burdett S., Sydes M.R. (2007). Practical methods for incorporating summary time-to-event data into meta-analysis. Trials.

[24] Doi S.A., Barendregt J.J., Khan S., Thalib L., Williams G.M. (2015). Advances in the meta-analysis of heterogeneous clinical trials I: The inverse variance heterogeneity model. Contemp. Clin. Trials.

[25] Friedrich J.O., Adhikari N.K., Beyene J. (2007). Inclusion of zero total event trials in meta-analyses maintains analytic consistency and incorporates all available data. BMC Med. Res. Methodol..

[26] Rothman K.J., Greenland S., Lash T.L. (2008). Modern Epidemiology.

[27] Higgins J.P.T., Thomas J., Chandler J., Cumpston M., Li T., Page M.J., Welch V.A. (2019). Cochrane Handbook for Systematic Reviews of Interventions Version 6.0.

[28] Higgins J.P., Thompson S.G., Deeks J.J., Altman D.G. (2003). Measuring inconsistency in meta-analyses. BMJ.

[29] Vicini F.A., Cecchini R.S., White J.R., Arthur D.W., Julian T.B., Rabinovitch R.A., Kuske R.R., Ganz P.A., Parda D.S., Scheier M.F. (2019). Long-term primary results of accelerated partial breast irradiation after breast-conserving surgery for early-stage breast cancer: A randomised, phase 3, equivalence trial. Lancet.

[30] Vicini F., Cecchini R., White J., Julian T., Arthur D., Rabinovitch R., Kuske R., Parda D., Ganz P., Scheier M. (2019). Abstract GS4-04: Primary results of NSABP B-39/RTOG 0413 (NRG Oncology): A randomized phase III study of conventional whole breast irradiation (WBI) versus partial breast irradiation (PBI) for women with stage 0, I, or II breast cancer. Cancer Res..

[31] White J.R., Winter K., Cecchini R.S., Vicini F.A., Arthur D.W., Kuske R.R., Rabinovitch R.A., Sehkon A., Khan A.J., Chmura S.J. (2019). Cosmetic Outcome from Post Lumpectomy Whole Breast Irradiation (WBI) Versus Partial Breast Irradiation (PBI) on the NRG Oncology/NSABP B39-RTOG 0413 Phase III Clinical Trial. Int. J. Radiat. Oncol. Biol. Phys..

[32] Whelan T.J., Julian J.A., Berrang T.S., Kim D.H., Germain I., Nichol A.M., Akra M., Lavertu S., Germain F., Fyles A. (2019). External beam accelerated partial breast irradiation versus whole breast irradiation after breast conserving surgery in women with ductal carcinoma in situ and node-negative breast cancer (RAPID): A randomised controlled trial. Lancet.

[33] Olivotto I.A., Whelan T.J., Parpia S., Kim D.H., Berrang T., Truong P.T., Kong I., Cochrane B., Nichol A., Roy I. (2013). Interim cosmetic and toxicity results from RAPID: A randomized trial of accelerated partial breast irradiation using three-dimensional conformal external beam radiation therapy. J. Clin. Oncol..

[34] Peterson D., Truong P.T., Parpia S., Olivotto I.A., Berrang T., Kim D.-H., Kong I., Germain I., Nichol A., Akra M. (2015). Predictors of Adverse Cosmetic Outcome in the RAPID Trial: An Exploratory Analysis. Int. J. Radiat. Oncol. Biol. Phys..

[35] Whelan T., Julian J., Levine M., Berrang T., Kim D.-H., Gu C., Germain I., Nichol A., Akra M., Lavertu S. (2019). Abstract GS4-03: RAPID: A randomized trial of accelerated partial breast irradiation using 3-dimensional conformal radiotherapy (3D-CRT). Cancer Res..

[36] Meattini I., Saieva C., Lucidi S., lo Russo M., Scotti V., Desideri I., Marrazzo L., Simontacchi G., Mangoni M., Becherini C. (2020). Abstract GS4-06: Accelerated partial breast or whole breast irradiation after breast conservation surgery for patients with early breast cancer: 10-year follow up results of the APBI IMRT Florence randomized phase 3 trial. Cancer Res..

[37] Livi L., Buonamici F.B., Simontacchi G., Scotti V., Fambrini M., Compagnucci A., Paiar F., Scoccianti S., Pallotta S., Detti B. (2010). Accelerated partial breast irradiation with IMRT: New technical approach and interim analysis of acute toxicity in a phase III randomized clinical trial. Int. J. Radiat. Oncol. Biol. Phys..

[38] Livi L., Meattini I., Marrazzo L., Simontacchi G., Pallotta S., Saieva C., Paiar F., Scotti V., De Luca Cardillo C., Bastiani P. (2015). Accelerated partial breast irradiation using intensity-modulated radiotherapy versus whole breast irradiation: 5-year survival analysis of a phase 3 randomised controlled trial. Eur. J. Cancer.

[39] Meattini I., Saieva C., Miccinesi G., Desideri I., Francolini G., Scotti V., Marrazzo L., Pallotta S., Meacci F., Muntoni C. (2017). Accelerated partial breast irradiation using intensity modulated radiotherapy versus whole breast irradiation: Health-related quality of life final analysis from the Florence phase 3 trial. Eur. J. Cancer.

[40] Vaidya J.S., Joseph D.J., Tobias J.S., Bulsara M., Wenz F., Saunders C., Alvarado M., Flyger H.L., Massarut S., Eiermann W. (2010). Targeted intraoperative radiotherapy versus whole breast radiotherapy for breast cancer (TARGIT-A trial): An international, prospective, randomised, non-inferiority phase 3 trial. Lancet.

[41] Andersen K.G., Gartner R., Kroman N., Flyger H., Kehlet H. (2012). Persistent pain after targeted intraoperative radiotherapy (TARGIT) or external breast radiotherapy for breast cancer: A randomized trial. Breast.

[42] Sperk E., Welzel G., Keller A., Kraus-Tiefenbacher U., Gerhardt A., Sutterlin M., Wenz F. (2012). Late radiation toxicity after intraoperative radiotherapy (IORT) for breast cancer: Results from the randomized phase III trial TARGIT A. Breast Cancer Res. Treat..

[43] Welzel G., Boch A., Sperk E., Hofmann F., Kraus-Tiefenbacher U., Gerhardt A., Suetterlin M., Wenz F. (2013). Radiation-related quality of life parameters after targeted intraoperative radiotherapy versus whole breast radiotherapy in patients with breast cancer: Results from the randomized phase III trial TARGIT-A. Radiat. Oncol..

[44] Keshtgar M.R., Williams N.R., Bulsara M., Saunders C., Flyger H., Cardoso J.S., Corica T., Bentzon N., Michalopoulos N.V., Joseph D.J. (2013). Objective assessment of cosmetic outcome after targeted intraoperative radiotherapy in breast cancer: Results from a randomised controlled trial. Breast Cancer Res. Treat..

[45] Corica T., Nowak A.K., Saunders C.M., Bulsara M., Taylor M., Vaidya J.S., Baum M., Joseph D.J. (2016). Cosmesis and Breast-Related Quality of Life Outcomes After Intraoperative Radiation Therapy for Early Breast Cancer: A Substudy of the TARGIT-A Trial. Int J. Radiat. Oncol. Biol. Phys..

[46] Corica T., Nowak A.K., Saunders C.M., Bulsara M.K., Taylor M., Williams N.R., Keshtgar M., Joseph D.J., Vaidya J.S. (2018). Cosmetic outcome as rated by patients, doctors, nurses and BCCT.core software assessed over 5 years in a subset of patients in the TARGIT-A Trial. Radiat. Oncol..

[47] Vaidya J.S., Bulsara M., Saunders C., Flyger H., Tobias J.S., Corica T., Massarut S., Wenz F., Pigorsch S., Alvarado M. (2020). Effect of Delayed Targeted Intraoperative Radiotherapy vs Whole-Breast Radiotherapy on Local Recurrence and Survival: Long-term Results From the TARGIT-A Randomized Clinical Trial in Early Breast Cancer. JAMA Oncol..

[48] Polgar C., Ott O.J., Hildebrandt G., Kauer-Dorner D., Knauerhase H., Major T., Lyczek J., Guinot J.L., Dunst J., Miguelez C.G. (2017). Late side-effects and cosmetic results of accelerated partial breast irradiation with interstitial brachytherapy versus whole-breast irradiation after breast-conserving surgery for low-risk invasive and in-situ carcinoma of the female breast: 5-year results of a randomised, controlled, phase 3 trial. Lancet Oncol..

[49] Schafer R., Strnad V., Polgar C., Uter W., Hildebrandt G., Ott O.J., Kauer-Dorner D., Knauerhase H., Major T., Lyczek J. (2018). Quality-of-life results for accelerated partial breast irradiation with interstitial brachytherapy versus whole-breast irradiation in early breast cancer after breast-conserving surgery (GEC-ESTRO): 5-year results of a randomised, phase 3 trial. Lancet Oncol..

[50] Bhattacharya I.S., Haviland J.S., Kirby A.M., Kirwan C.C., Hopwood P., Yarnold J.R., Bliss J.M., Coles C.E., Trialists I. (2019). Patient-Reported Outcomes Over 5 Years After Whole- or Partial-Breast Radiotherapy: Longitudinal Analysis of the IMPORT LOW (CRUK/06/003) Phase III Randomized Controlled Trial. J. Clin. Oncol..

[51] Bhattacharya I.S., Haviland J.S., Hopwood P., Coles C.E., Yarnold J.R., Bliss J.M., Kirby A.M., Trialists I. (2019). Can patient-reported outcomes be used instead of clinician-reported outcomes and photographs as primary endpoints of late normal tissue effects in breast radiotherapy trials? Results from the IMPORT LOW trial. Radiother. Oncol..

[52] Bhattacharya I.S., Haviland J.S., Perotti C., Eaton D., Gulliford S., Harris E., Coles C.E., Kirwan C.C., Bliss J.M., Kirby A.M. (2019). Is breast seroma after tumour resection associated with patient-reported breast appearance change following radiotherapy? Results from the IMPORT HIGH (CRUK/06/003) trial. Radiother. Oncol..

[53] Polgar C., Major T., Fodor J., Nemeth G., Orosz Z., Sulyok Z., Udvarhelyi N., Somogyi A., Takacsi-Nagy Z., Lovey K. (2004). High-dose-rate brachytherapy alone versus whole breast radiotherapy with or without tumor bed boost after breast-conserving surgery: Seven-year results of a comparative study. Int. J. Radiat. Oncol. Biol. Phys..

[54] Polgar C., Fodor J., Major T., Nemeth G., Lovey K., Orosz Z., Sulyok Z., Takacsi-Nagy Z., Kasler M. (2007). Breast-conserving treatment with partial or whole breast irradiation for low-risk invasive breast carcinoma-5-year results of a randomized trial. Int. J. Radiat. Oncol. Biol. Phys..

[55] Offersen B., Nielsen H.M., Thomsen M., Jakobsen E., Nielsen M.H., Stenbygaard L., Pedersen A.N., Krause M., Jensen M.-B., Overgaard J. (2017). SP-0315: Partial breast radiotherapy after breast conservation for breast cancer: Early results from the randomised DBCG PBI trial. Radiother. Oncol..

[56] Haviland J.S., Owen J.R., Dewar J.A., Agrawal R.K., Barrett J., Barrett-Lee P.J., Dobbs H.J., Hopwood P., Lawton P.A., Magee B.J. (2013). The UK Standardisation of Breast Radiotherapy (START) trials of radiotherapy hypofractionation for treatment of early breast cancer: 10-year follow-up results of two randomised controlled trials. Lancet Oncol..

[57] Dell’Oro M., Giles E., Sharkey A., Borg M., Connell C., Bezak E. (2019). A Retrospective Dosimetric Study of Radiotherapy Patients with Left-Sided Breast Cancer; Patient Selection Criteria for Deep Inspiration Breath Hold Technique. Cancers.

[58] Jagsi R., Griffith K.A., Moran J.M., Ficaro E., Marsh R., Dess R.T., Chung E., Liss A.L., Hayman J.A., Mayo C.S. (2018). A Randomized Comparison of Radiation Therapy Techniques in the Management of Node-Positive Breast Cancer: Primary Outcomes Analysis. Int. J. Radiat. Oncol. Biol. Phys..

[59] Duma M.N., Baumann R., Budach W., Dunst J., Feyer P., Fietkau R., Haase W., Harms W., Hehr T., Krug D. (2019). Heart-sparing radiotherapy techniques in breast cancer patients: A recommendation of the breast cancer expert panel of the German society of radiation oncology (DEGRO). Strahlenther. Onkol..

[60] Cuzick J., Stewart H., Peto R., Baum M., Fisher B., Host H., Lythgoe J.P., Ribeiro G., Scheurlen H., Wallgren A. (1987). Overview of randomized trials of postoperative adjuvant radiotherapy in breast cancer. Cancer Treat. Rep..

[61] Hickey B.E., Lehman M., Francis D.P., See A.M. (2016). Partial breast irradiation for early breast cancer. Cochrane Database Syst. Rev..

[62] Correa C., Harris E.E., Leonardi M.C., Smith B.D., Taghian A.G., Thompson A.M., White J., Harris J.R. (2017). Accelerated Partial Breast Irradiation: Executive summary for the update of an ASTRO Evidence-Based Consensus Statement. Pract. Radiat. Oncol..

[63] Strnad V., Major T., Polgar C., Lotter M., Guinot J.L., Gutierrez-Miguelez C., Galalae R., Van Limbergen E., Guix B., Niehoff P. (2018). ESTRO-ACROP guideline: Interstitial multi-catheter breast brachytherapy as Accelerated Partial Breast Irradiation alone or as boost—GEC-ESTRO Breast Cancer Working Group practical recommendations. Radiother. Oncol..

[64] Shah C., Vicini F., Shaitelman S.F., Hepel J., Keisch M., Arthur D., Khan A.J., Kuske R., Patel R., Wazer D.E. (2018). The American Brachytherapy Society consensus statement for accelerated partial-breast irradiation. Brachytherapy.

[65] Strnad V., Hannoun-Levi J.-M., Guinot J.-L., Lössl K., Kauer-Dorner D., Resch A., Kovács G., Major T., Van Limbergen E. (2015). Recommendations from GEC ESTRO Breast Cancer Working Group (I): Target definition and target delineation for accelerated or boost Partial Breast Irradiation using multicatheter interstitial brachytherapy after breast conserving closed cavity surgery. Radiother. Oncol..

[66] Strnad V., Krug D., Sedlmayer F., Piroth M.D., Budach W., Baumann R., Feyer P., Duma M.N., Haase W., Harms W. (2020). DEGRO practical guideline for partial-breast irradiation. Strahlenther. Onkol..

